# Identifying time-delayed gene regulatory networks via an evolvable hierarchical recurrent neural network

**DOI:** 10.1186/s13040-017-0146-4

**Published:** 2017-08-03

**Authors:** Mina Moradi Kordmahalleh, Mohammad Gorji Sefidmazgi, Scott H. Harrison, Abdollah Homaifar

**Affiliations:** 1 Department of Electrical and Computer Engineering, North Carolina A&T State University, 1601 E. Market Street, Greensboro, 27411 NC USA; 2 Department of Biology, North Carolina A&T State University, 1601 E. Market Street, Greensboro, 27411 NC USA

**Keywords:** Gene regulatory network, Hierarchical recurrent neural network, Genetic algorithm, Time delay

## Abstract

**Background:**

The modeling of genetic interactions within a cell is crucial for a basic understanding of physiology and for applied areas such as drug design. Interactions in gene regulatory networks (GRNs) include effects of transcription factors, repressors, small metabolites, and microRNA species. In addition, the effects of regulatory interactions are not always simultaneous, but can occur after a finite time delay, or as a combined outcome of simultaneous and time delayed interactions. Powerful biotechnologies have been rapidly and successfully measuring levels of genetic expression to illuminate different states of biological systems. This has led to an ensuing challenge to improve the identification of specific regulatory mechanisms through regulatory network reconstructions. Solutions to this challenge will ultimately help to spur forward efforts based on the usage of regulatory network reconstructions in systems biology applications.

**Methods:**

We have developed a hierarchical recurrent neural network (HRNN) that identifies time-delayed gene interactions using time-course data. A customized genetic algorithm (GA) was used to optimize hierarchical connectivity of regulatory genes and a target gene. The proposed design provides a non-fully connected network with the flexibility of using recurrent connections inside the network. These features and the non-linearity of the HRNN facilitate the process of identifying temporal patterns of a GRN.

**Results:**

Our HRNN method was implemented with the Python language. It was first evaluated on simulated data representing linear and nonlinear time-delayed gene-gene interaction models across a range of network sizes and variances of noise. We then further demonstrated the capability of our method in reconstructing GRNs of the *Saccharomyces cerevisiae* synthetic network for in vivo benchmarking of reverse-engineering and modeling approaches (IRMA). We compared the performance of our method to TD-ARACNE, HCC-CLINDE, TSNI and ebdbNet across different network sizes and levels of stochastic noise. We found our HRNN method to be superior in terms of accuracy for nonlinear data sets with higher amounts of noise.

**Conclusions:**

The proposed method identifies time-delayed gene-gene interactions of GRNs. The topology-based advancement of our HRNN worked as expected by more effectively modeling nonlinear data sets. As a non-fully connected network, an added benefit to HRNN was how it helped to find the few genes which regulated the target gene over different time delays.

## Background

New opportunities to reverse engineer the activities of different components of complex cellular systems are arising due to technologies like DNA microarrays and RNA sequencing which provide genomic-scale data sets [1, 2]. Time series data may be collected either in longitudinal studies of cell or tissue samples collected over multiple time points [3], or expressional change across state space [4]. Effective models of gene regulatory networks (GRNs) have successfully identified regulatory interactions between genes and the specific functional roles of individual genes in cellular systems [5, 6].

**Fig. 1:**
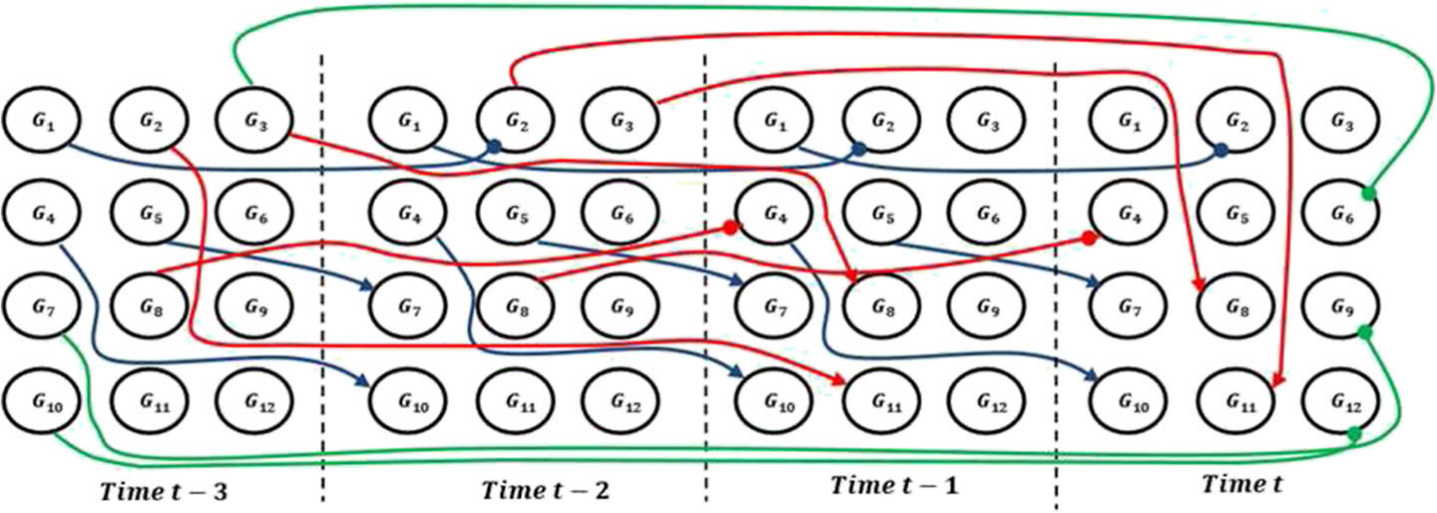
Time-delayed regulatory interactions among 12 genes with maximum time lag equal to 3. Colors *black*, *red* and *green* represent time delays of one, two and three steps

Reverse engineering of GRNs occurs within the context of stochastic properties of the system, measurement noise, and high dimensionality [3]. There is strong non-linearity on temporal patterns of regulatory genes [7]. Further complexity ensues given that genetic interactions among different genes can have different time delays [8, 9]. These delays are due to the transcription and translation of genes varying in composition and length, along with varying kinetics of binding and completion with respect to genes being processed by the transcriptome, the spliceosome and the ribosome. Transcribed and translated products may be further converted and are eventually degraded, with some products being more stable than others. Changing physiological conditions can impact many of the above factors of time delays. As shown in Fig. 1, there are complex combinations by which the expression level of a gene at a certain time could depend upon the expression level of another gene at a previous time point.

Diverse methods with different levels of complexity have been used to model, analyze and infer complex regulatory interactions [10–13]. Boolean networks are the simplest among them [14]. They are based only upon binary outcomes (on and off) for gene expression and therefore lack adequate dynamic resolution. Bayesian networks represent probabilistic relationships among genes and have shown some success in capturing the inherent noise and stochasticity of gene expression data [15]. Dynamic Bayesian Networks (DBN) are an extension of Bayesian networks that can unravel the feedback cycles and loops over time points [16]. However, due to their high computational cost, the application of dynamic Bayesian networks is limited to small networks. Ordinary Differential Equations (ODE) are deterministic models, where interactions among genes represent causal interactions rather than statistical dependencies [17]. They can offer continuous representations of genetic networks, but are not robust for imprecise data.

Methods such as time delay linear regression [18], correlation matrices [19], stochastic simulation algorithms [9, 20], dynamic Bayesian networks [16] and delayed differential equations [21] have been proposed to incorporate a fixed time delay in GRN models. In [22], pairwise correlations between each pair of genes have been used to address the various time delays in gene interactions. TD-ARACNE (Time Delay-Algorithm for the Reconstruction of Accurate Cellular Networks) has been proposed in [23]. This algorithm detects the time-delayed dependencies between the expression profiles in terms of mutual information by assuming a stationary Markov Random Field as its underlying probabilistic model. The TD-ARACNE algorithm does not assign any specific delay or regulatory effect on the edges of the GRN. HCC-CLINDE [24] is an extension of CLINDE [25], and has been developed to infer a time-delayed GRN in the presence of hidden common causes. All directed pairs of genes in the network have possible delays up to a maximum allowed delay, which is obtained based on either a correlation test or mutual information test.

The main objective of this paper is to reconstruct a time-delayed GRN which takes into account the non-linearity of gene interaction and the noise of temporal measurements. Recurrent Neural Networks (RNNs) are computational tools inspired by the structural and functional aspects of biological nervous systems, and are noted for their effectiveness in temporal data processing and approximating nonlinear patterns of dynamic temporal behaviors [26]. The ability of RNNs to learn from temporal data, estimate multivariate nonlinear functions, and tolerate noise in measurements makes RNNs an ideal fit for the modeling of gene regulatory interactions using gene expression profiles. Several variants of RNNs have been deployed for the modeling of GRNs including neural fuzzy recurrent networks [27], RNNs combined with particle swarm optimization [28], ensemble of RNNs and support vector machines [29], RNNs combined with differential evolution [30] and RNNs hybridized with the generalized extended Kalman filter [31]. Despite the great capabilities of RNNs for predictive modeling with high accuracy, RNNs are usually considered “black box” models whose internal structure and learned parameters are not interpretable. Due to the multiple layers, the non-linearity of the model, and cyclic (feedback) connections in the network structure, their interpretability still remains vague [32]. This, in particular, impedes goals with GRN reconstruction to identify pairs of genes, directions of regulation, effects (i.e. up or down regulation), and time delays.

In this paper, we have proposed a hierarchical RNN (HRNN) that surmounts the interpretation difficulties of the RNNs for application of GRN modeling. The proposed design lets us use the features of hierarchical representation in addition to the capabilities of RNNs for finding temporal dependencies. In this way, time-delayed regulations can be captured through hierarchical paths between leaf nodes (regulatory genes) and a target node (regulated gene) in the HRNN. For discovering the underlying hierarchical structure among the regulatory genes and a target gene, the network topology and connection weights are encoded by a customized genetic algorithm (GA). Through the training procedure, in addition to evolving network connection weights, the GA rewires the connectivity and length of hierarchical paths between leaf nodes and the target gene of a population of candidate networks. From the trained HRNN, the direction and effect of gene regulations in the presence of time delays can be captured. Our proposed model is evaluated on a real biological system and linear/nonlinear synthetic generated data for different sizes of networks and variances of noise. The results of our HRNN method are compared with TD-ARACNE, HCC-CLINDE, ODE implemented in the TSNI package [33] and ebdbNet package (Empirical Bayes Dynamic Bayesian Network Inference) [34].

## Method

Assume that $\{G_{1}^{l}(t), G_{2}^{l}(t),\ldots, G_{P}^{l}(t)\}$ are expression levels of *P* genes at time *t* in experiment *l* where *t*∈{1,…,*T* ^*l*^} and *l*∈{1,…,*L*}. The aim is to capture the potential regulators for each gene *G*_*i*_ in a decoupled hierarchical RNN. In this network, *G*_*i*_ is the target gene and the rest of the genes are the potential regulators. At the beginning, a population of candidate hierarchical RNNs should be randomly generated. A candidate network can have 0≤*c*≤*C* context nodes in its structure. The network with *c* context nodes has *c*+1 neurons. Neurons are the processing units in the RNNs which induce non-linearity on the inputs. Neurons have multiple inputs and one output. The maximum number of context nodes in the candidate networks is set to *C*. Context nodes in the hierarchical RNN are nodes without experimental measurements and assist with modeling of temporal dynamics.

Assume that *x*_1_,…,*x*_*C*_ are context nodes and *x*_*C*+1_,…,*x*_*C*+*P*_ are genes. In a network with *c*≤*C* context nodes, the first *c* context nodes *x*_1_,…,*x*_*c*_ and genes (excluded the target gene) are potential inputs of the *c*+1 neurons in the network. In each candidate network, the target gene is the output of the first neuron, and the context node *c*_*i*_ is the input of *neuron*_*i*_ and output of *neuron*_*i*+1_ where *i*∈{1,…,*c*}. In addition to the genes and context node *c*_*i*_, other context nodes could also be the potential inputs of the neuron *neuron*_*i*+1_, except for *neuron*_*c*+1_, which has no context nodes as its inputs. Each input connection has a weight. If context node is the input of the neuron, the corresponding connection weight is positive. Else, it could be positive or negative. Through training, the customized GA evolves the connectivity between nodes and neurons and connection weights.

**Fig. 2:**
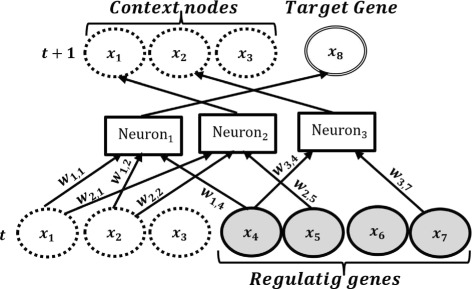
The structure of a hierarchical RNN with 2 context variables, 5 genes and 3 neurons. Each neuron has only one outgoing connection. For example, *Neuron*_2_ has three incoming connections *x*_1_,*x*_2_,*x*_5_ at time *t* with corresponding weights *w*_2,1_,*w*_2,2_,*w*_2,5_ and an outgoing connection to context node *x*_1_ at time *t*+1. Context nodes, regulatory genes and the target gene are shown by broken, highlighted and double-line ovals respectively

**Fig. 3:**
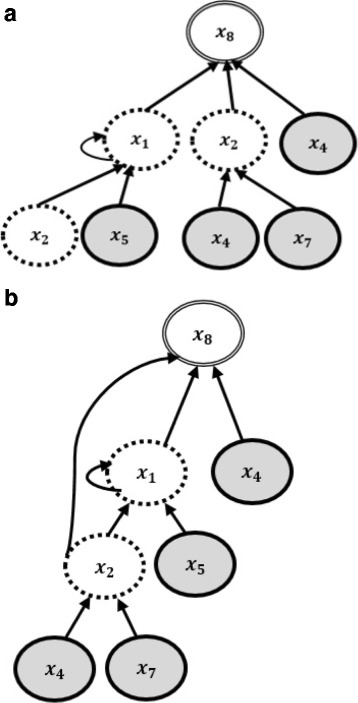
The corresponding hierarchical model which shows the direct regulation of *x*_8_ by gene *x*_4_, and time-delayed regulations of *x*_8_ by genes *x*_4_,*x*_5_,*x*_7_. Context nodes, regulatory genes and the target gene are shown by broken, highlighted and double-line ovals respectively. **a** The corresponding hierarchical recurrent structure obtained from Fig. 2. **b** Regulation of the target gene *x*_8_ with connections from context nodes *x*_1_ and *x*_2_

Figure 2 shows a candidate network generated from a maximum possible number of three context nodes (*x*_1_,*x*_2_,*x*_3_) and five genes (*x*_4_,*x*_5_,*x*_6_,*x*_7_,*x*_8_). The candidate network in this figure uses two out of the three possible context nodes; thus it has three neurons. This figure shows the regulatory interactions of target gene *x*_8_ with *x*_4_,*x*_5_ and *x*_7_. Figure 3(a) shows the corresponding hierarchical recurrent structure obtained from Fig. 2. For Fig. 3(b), time-delayed regulations of the target gene *x*_8_ are captured by *x*_4_,*x*_5_,*x*_7_ in presence of two context nodes *x*_1_ and *x*_2_.

For the target and context nodes *x*_*i*_ that are outputs of the neurons, Eq. 1 shows the updated value from time *t* to *t*+1.

1 $$\begin{array}{@{}rcl@{}} x_{i}(t+1)=f\left(\sum\limits_{j} w_{k,j}x_{j}(t)\right) \end{array} $$

where *x*_*j*_(*t*) is the value of *j* ^*th*^ input node connected to *x*_*i*_(*t*+1). *f* is a sigmoid function in the form of Eq. 2 which is monotonically increasing in the range of [0,1] and is commonly used in the literature to induce non-linearity.

2 $$\begin{array}{@{}rcl@{}} f(x)=\frac{1}{{1+{e^{-(x)}}}} \end{array} $$

In the case of self-regularization of the target gene, Eq. 3 is used for updating the value of the target gene:

3 $$\begin{array}{@{}rcl@{}} x_{i}(t+1)=f\left(\sum\limits_{j}w_{k,j}x_{j}(t)\right)-\mu x_{i}(t) \end{array} $$

where *μ* is the decay rate of the target gene’s expression over time. Estimating the decay rate for each gene helps to model the suppression effect of a gene on itself [35].

### Evolutionary training algorithm

A customized GA is proposed for training the HRNN. At the beginning, the GA generates a population of random candidate networks. The structure and connection weights of the candidate networks are evolved over generations of the GA with the guidance of the fitness function. In each generation, new candidate networks (children) are formed by applying the evolutionary operators (crossover and mutation) on the old networks (parents) within the constraints of the HRNN. Parents are selected according to their fitness values, where networks with higher accuracy have more chances to reproduce. The newly generated population is used for the next generation of the GA. At each generation, an elitist evolving strategy is applied to keep the best candidate networks from the last population. The evolutionary process is repeated until the terminating conditions are satisfied. The proposed procedure is summarized in Algorithm ??.



#### Representation of candidate networks

**Fig. 4:**
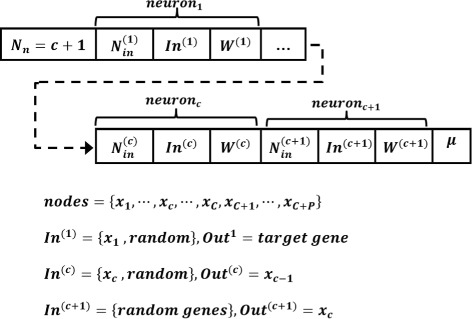
Representation of the candidate hierarchical RNNs in the GA. This diagram displays the number of neurons in the network along with their connectivity description

Candidate networks in the GA are represented by their number of neurons (*N*_*n*_), number of inputs to each neuron (*N*_*in*_), indices of the input nodes (*In*), weights of the input connections (*W*) and the decay rate of the target gene’s expression level (*μ*) if it exists. Components of the first, *c* ^*th*^ and last neurons in a candidate network with *c*+1 neurons are represented in Fig. 4. In this candidate network, *P* genes and *c* out of *C* context nodes are used in the network. One of the genes is considered as a target gene. The output of each neuron (*Out*) does not change in the training process.

#### Fitness of candidate networks

The performance of the candidate networks (fitness) is evaluated by measuring the trade-off between the goodness of fit and complexity of the model by using the Akaike information criterion (AIC) and the Akaike information criterion with correction (AICc). AIC is a model selection criterion which estimates the quality of a model relative to other models (Eq. 4).

4 $$\begin{array}{@{}rcl@{}} AIC=n\left[ln \left(\frac{\sum_{l}\sum_{t} \left(x_{i}^{l}(t)-\hat{x}_{i}^{l}(t)\right)^{2}}{n}\right)\right]+2k \end{array} $$

where $x_{i}^{l}(t)$ is the expression value of the target gene *i* at experiment *l*, and $\hat {x}_{i}^{l}$ is the corresponding estimation by the candidate HRNN at time *t*. *k* is the number of leaf nodes in the HRNN and *n* is the total number of temporal samples for gene expression. If *n* is small or *k* is large, the AICc is preferred rather than AIC (Eq. 5). As *n* gets larger, AICc converges to AIC.

5 $$\begin{array}{@{}rcl@{}} AICc=AIC+ \frac{2k(k+1)}{n-k-1} \end{array} $$

#### Crossover operator

Crossover is an evolutionary operator for generating a new candidate network. Before applying the proposed crossover, the tournament selection reproduces a new pool of candidate networks. The tournament selection is a method of selecting a candidate among a few candidates chosen at random from the population. The winner of each tournament (the one with the best fitness) will be replaced in the new pool.

**Fig. 5:**
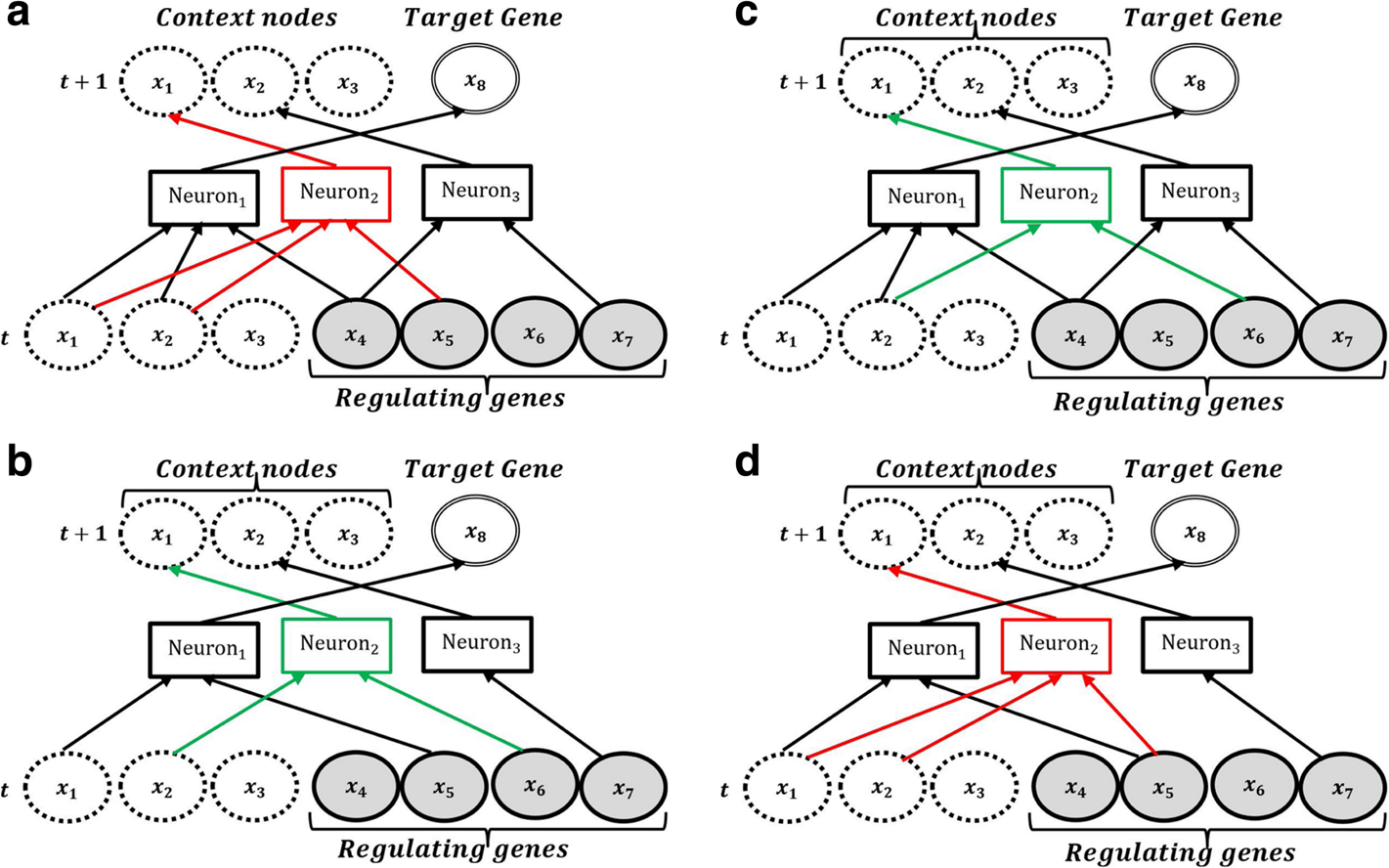
The crossover operator swaps the input/output connections of the second neurons in the parent 1 and 2. The new generated networks are cross-children 1 and 2. **a** Parent 1 **b** Parent 2 **c** Cross-children 1 **d** Cross-children 2

For applying the proposed crossover, first the networks in the population are shuffled and sorted by the number of neurons (*N*_*n*_) in their structure. Then, for each pair of selected networks with the same number of neurons (parents 1 and 2), crossover with probability of *P*_*c*_ swaps the random neurons *i*∈{1,…,*N*_*n*_} in two parents. Figure 5(c)-(d) show the crossover operating on neuron 2 of the parents in Figure 5(a)-(b). Crossover creates new candidate networks (cross-children) with new connectivity and connection weights.

#### Mutation operator

The mutation operator mutates the number of inputs of the neurons, rewires the connectivity of the inputs of the neurons, and evolves the connection weights with the probability *P*_*m*_. For a mutation site *m*_*site*_ in the network, the mutation works as below:

- If *m*_*site*_ is on the number of inputs of a neuron (*N*_*in*_), it is mutated to *N*_*in*_=*N*_*in*_±1. Therefore, a new input and its corresponding weight are added or deleted.
- If *m*_*site*_ is on an input connection of a neuron (*In*), the selected connection is rewired to another node in the network.
- If *m*_*site*_ is on a connection weight of a neuron and input is a context node, the Gaussian mutation evolves the weight in the range of [0,*w*_*max*_]; else, the weight is mutated in the range of [*w*_*min*_,*w*_*max*_]
- If *m*_*site*_ is on the decay rate *μ*, the Gaussian mutation is applied to evolve the the decay rate in the range [0,*w*_*max*_].

## Experimental results

In order to evaluate the performance of the proposed method, we have tested our method with both synthetic data and real data against TD-ARACNE, HCC-CLINDE, TSNI and ebdbNet. As the underlying regulatory networks for the real biological datasets are generally unknown, synthetic data are helpful for checking the efficiency of methods. The generated synthetic models in this paper have different levels of complexity and enable us to have a broad-ranging performance evaluation of our proposed approach in comparison to other approaches. In a real life experiment, we applied our method for finding the GRN of *Saccharomyces cerevisiae*.

We assess the performance of the inference algorithm on three aspects, namely Links (which is considered correct if and only if both the gene pair and the direction are correct), Delays (which is considered correct if and only if both the link and the time delay are correct), and Effects (which is considered correct if and only if both the link and the sign of an effect are correct) [24]. For each aspect, Recall $=\frac {TP}{TP+FN}$, Precision $=\frac {TP}{TP+FP}$ and *F*-score $=\frac {2 \times Precision \times Recall}{Precision+Recall}$ metrics are computed. In these metrics, *TP, FP* and *FN* are numbers of true positives, false positives, and false negatives respectively. The *F*-scores of the results have been compared as an overall measurement of performance. The HCC-CLINDE method provides *F*-scores of Link, Delay and Effect criteria. However, TD-ARACNE, TSNI and ebdbNet provide information for finding the *F*-score of the Link. In all simulations, algorithms have been tested by their default parameters.

### Synthetic data

The generation of synthetic data has been considered in two instances of linear and nonlinear models. To compare the accuracy of the HRNN with TD-ARACNE, HCC-CLINDE, TSNI and ebdbNet, the effects of the noise levels and number of genes in small and medium size networks are investigated. For a chosen number of genes and level of noise, ten random GRNs with random connectivity between nodes, weight of the connections, time delay and initial value of gene expressions are generated. The purpose of these experiments is to evaluate the performance of the proposed method in terms of linearity versus non-linearity of gene expression values, network size (*P*∈{5,10,20,30}) and noise variance (*σ* ^2^∈{0.1,0.25,0.5,1.0,1.5}). The linear and nonlinear models of random GRNs are generated by Eqs. 6 and 7 respectively:

6 $$\begin{array}{@{}rcl@{}} G_{i}(t+1)=\sum\limits_{j} a_{i,j}G_{j}(t-\tau_{ij})+\varepsilon_{i}(t) \end{array} $$

7 $$\begin{array}{@{}rcl@{}} G_{i}(t+1)=\sum\limits_{j} f(a_{i,j}G_{j}(t-\tau_{ij}))+\varepsilon_{i}(t) \end{array} $$

where *G*_*i*_(*t*) is the expression value of gene *i* at time *t*, *i*∈{1,…,*P*} and *t*∈{1,…,50}. *τ*_*ij*_∈{1,…,*τ*_*max*_} is the time delay of the edge *j*→*i* (if *τ*_*ij*_≠0). *a*_*ij*_ is the regulatory effect of gene *j* on gene *i*, where the regulatory effect is repressive if *a*_*ij*_ is negative, activatory if positive, and absent if zero. *ε*_*i*_(*t*) is a random Gaussian noise with zero mean and variance *σ* ^2^. The regulatory effects *a*_*ij*_ are randomly selected at the beginning of each simulation run. The network generation algorithm is set in such a way that each gene could have a maximum of 3 regulators with maximum delay *τ*_*max*_=4. The number of regulators and time lag for each edge in the synthetic networks are also generated randomly. In Eq. 7, *f* is a sigmoid and monotonically increasing function in the form of Eq. 2 which adds non-linearity to the model.

**Fig. 6:**
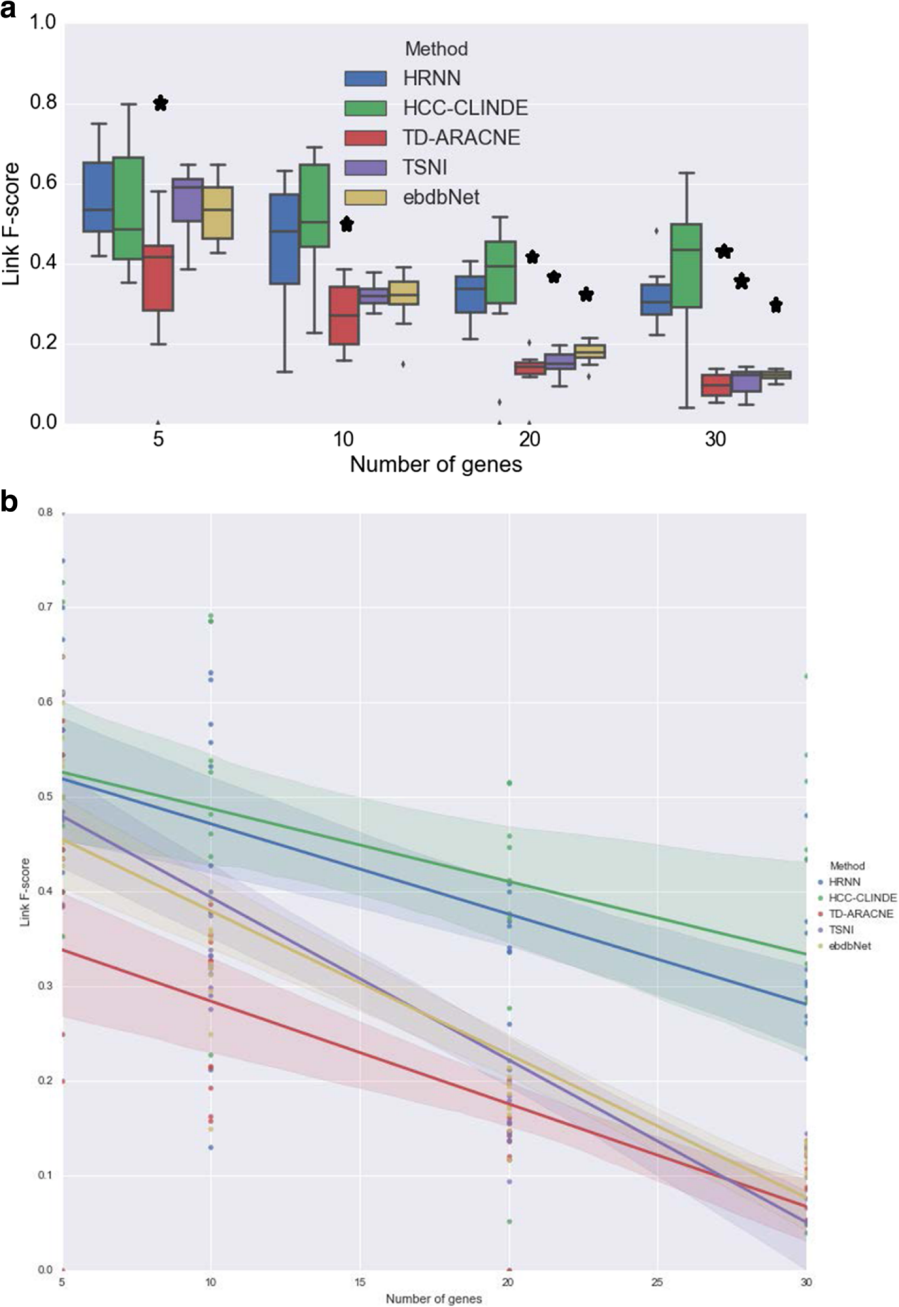
Effect of number of genes on Link *F*-score for case of linearity among gene interactions. For a chosen number of genes, 10 GRNs are randomly generated where *Var*(*noise*)=1. **a** Box plot of the Link *F*-score versus number of genes. ⋆ adjusted *p*-value ≤0.05**b** Linear regression model of the Link *F*-score versus number of genes

**Fig. 7:**
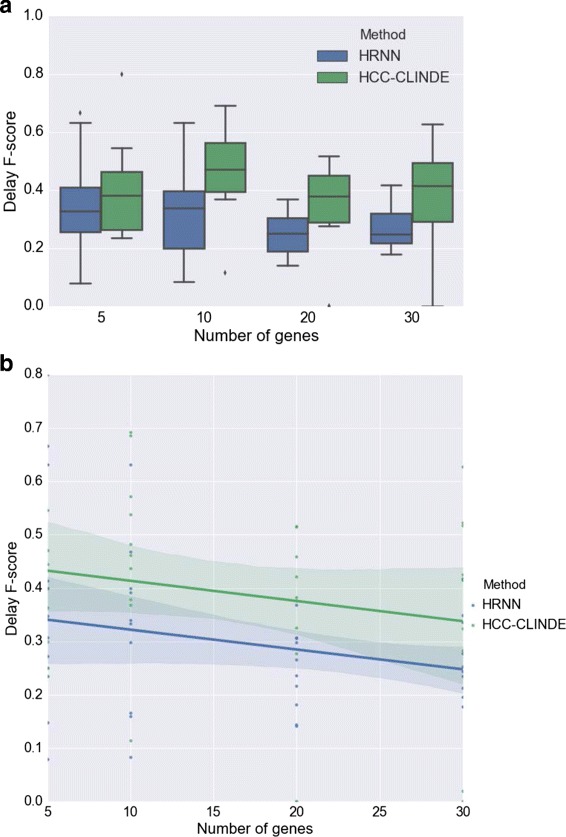
Effect of number of genes on Delay *F*-score for case of linearity among gene interactions. For a chosen number of genes, 10 GRNs are randomly generated where *Var*(*noise*)=1. **a** Box plot of the Delay *F*-score versus number of genes. ⋆ adjusted *p*-value ≤0.05**b** Linear regression model of the Delay *F*-score versus number of genes

**Fig. 8:**
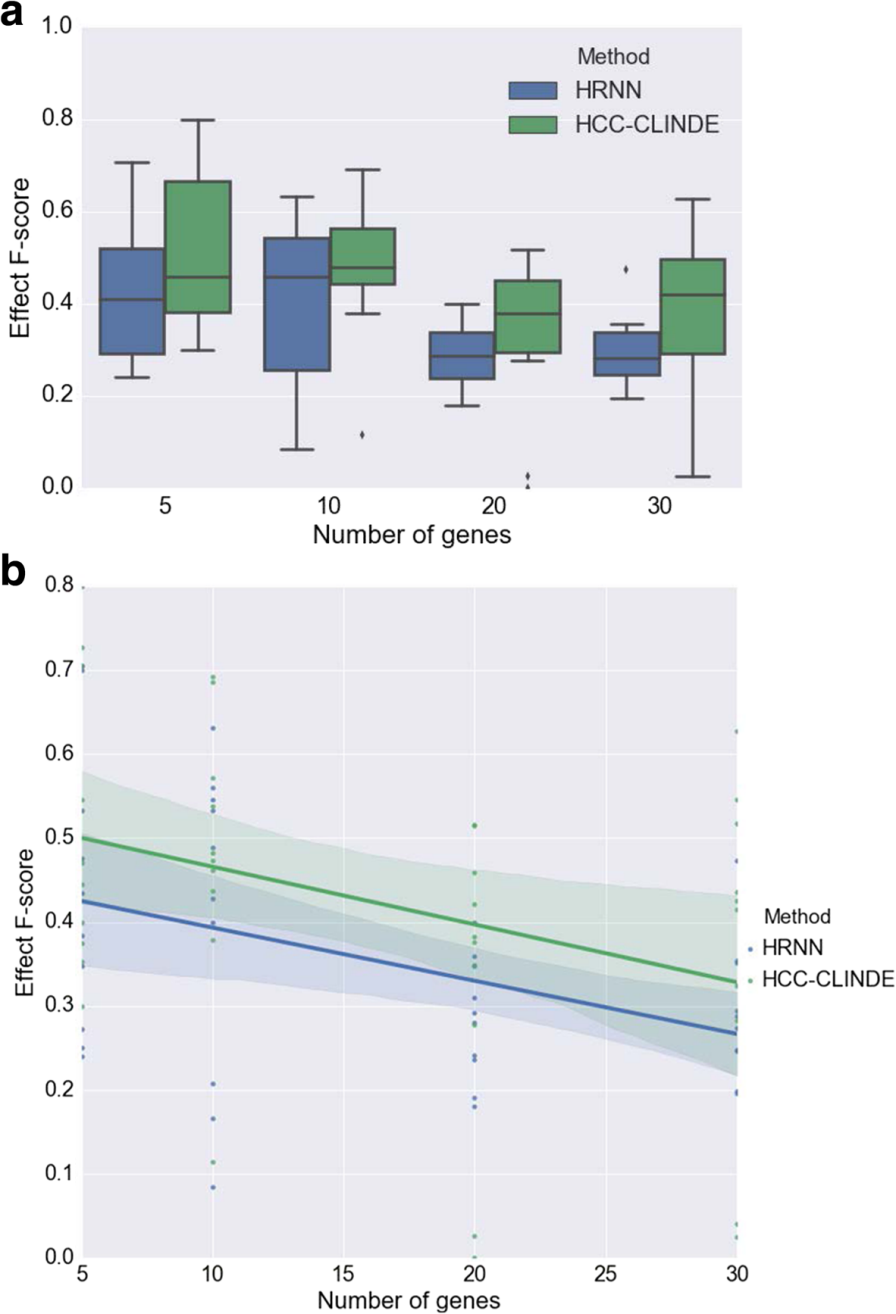
Effect of number of genes on Effect *F*-score for case of linearity among gene interactions. For a chosen number of genes, 10 GRNs are randomly generated where *Var*(*noise*)=1. **a** Box plot of the Effect *F*-score versus number of genes. ⋆ adjusted *p*-value ≤0.05**b** Linear regression model of the Effect *F*-score versus number of genes

The accuracy of GRN reconstruction using synthetic gene expression data generated from the linear model are presented in Figs. 6, 7 and 8. These figures compare the Link *F*-score, Delay *F*-score and Effect *F*-score of the HRNN with HCC-CLINDE, TD-ARACNE, TSNI and ebdbNet for different number of genes in the network. The variance of noise in these experiments is the same, and is equal to (*σ* ^2^=1). In part *a* of these figures, the box plot of *F*-score values of Link, Delay and Effect criterion are compared. In parts *b* of these figures, a linear regression model is fit on the *F*-score values with respect to the number of genes in the network. *R*-squared and *p*-values of the linear regression models in Figs. 6(b), 7(b) and 8(b) are stated in Table 1. In case of the linear data, the linear regression models in Fig. 6(b) shows that the HRNN and the HCC-CLINDE are responsibly competitive for finding the correct Link between the nodes in networks.

**Table 1:** The statistical properties of the linear regression models fitted on the *F*-score values with respect to the number of genes. Gene expression data is generated from a linear model

	Method	*R*-squared	*p*-value
Link	HRNN	0.38	2.3×10^−5^
	HCC-CLINDE	0.16	0.01
	TD-ARACNE	0.51	2.0×10^−7^
	TSNI	0.79	1.8×10^−14^
	ebdbNet	0.76	1.2×10^−13^
Delay	HRNN	0.06	0.10
	HCC-CLINDE	0.03	0.22
Effect	HRNN	0.16	0.008
	HCC-CLINDE	0.12	0.02

Table 2 includes the average of the TP, FP, FN, Precision, Recall and *F*-score of Link criteria for 10 independent runs of the methods and different number of genes in the network. Also, we conduct a hypothesis test for the difference between means of *F*-score. For a selected number of genes in Table 2, a *t*-test performed on *F*-score values of the HRNN and other methods. The null hypothesis is defined as two population means are equal. The nominal and adjusted *p*-values are mentioned in the table. HRNN is tested multiple times for four different number of genes in the network, to obtain the adjusted *p*-values, the nominal *p*-values are multiplied by four. If the corresponding adjusted *p*-value is less than 0.05, the null hypothesis is rejected, meaning that mean of *F*-scores are significantly different. *p*-values are for how the *F*-scores of the other methods may significantly differ with the *F*-scores of HRNN; therefore, *p*-values are not shown for the HRNN row in the tables. Table 3 includes the average of the TP, FP, FN, Precision, Recall, *F*-score and also *p*-values of Delay and Effect criterion for 10 independent runs of the HRNN and HCC-CLINDE and different number of genes in the network. Results shows that HRNN and HCC-CLINDE are not significantly different in terms of Delay and Effect *F*-scores in case of linearity among genes.

**Table 2:** The effect of network size on GRN reconstruction in case of linearity between the genes. Results are the average of the accuracy in terms of the Link criteria for GRN reconstruction of 10 different randomly generated synthetic networks

Methods	*P*	TP	FP	FN	Precision	Recall	*F*-score	Nominal *p*-value	Adjusted *p*-value
HRNN	5	5.6	4.5	4.3	0.56	0.57	0.56		
	10	9.7	15.2	9.6	0.39	0.50	0.44		
	20	14.8	36.5	25.3	0.28	0.37	0.32		
	30	21.8	58.1	36.5	0.27	0.37	0.31		
TD-ARACNE	5	4.3	7.2	5.6	0.33	0.43	0.36	0.008	0.03
	10	7.8	27.2	11.5	0.22	0.39	0.26	0.01	0.04
	20	9.1	76.9	31.0	0.10	0.23	0.13	1.6×10^−6^	6.4×10^−6^
	30	11.3	153.7	47.0	0.07	0.19	0.09	5.0×10^−8^	2.0×10^−7^
HCC-CLINDE	5	4.2	2.1	5.7	0.70	0.44	0.53	0.65	1
	10	8.4	5.5	10.9	0.62	0.43	0.51	0.35	1
	20	11.9	18.5	28.2	0.40	0.29	0.34	0.77	1
	30	17.7	19.8	40.6	0.48	0.30	0.37	0.44	1
TSNI	5	9.9	15.1	0.0	0.39	1.0	0.56	0.99	1
	10	17.9	73.1	1.4	0.19	0.93	0.32	0.04	0.16
	20	19.5	188.5	20.6	0.09	0.48	0.15	1.1×10^−6^	4.4×10^−6^
	30	14.3	189.7	44.0	0.06	0.24	0.10	1.2×10^−7^	4.8×10^−7^
ebdbNet	5	7.8	11.2	2.1	0.40	0.77	0.53	0.47	1
	10	15.8	63.2	3.5	0.19	0.80	0.31	0.03	0.12
	20	27.4	234.6	12.7	0.10	0.66	0.17	7.6×10^−6^	2.8×10^−5^
	30	31.5	430.5	26.8	0.06	0.53	0.12	9.7×10^−8^	3.6×10^−7^

The *p*-values are for how the Link *F*-scores of other methods compare with HRNN. *P* is the number of genes for each of the networks. The variance of the noise is equal to 1

**Table 3:** The effect of network size on GRN reconstruction in case of linearity between the genes. Results are the average of the accuracy in terms of the Delay and Effect criterion for GRN reconstruction of 10 different randomly generated synthetic networks

	Methods	*P*	TP	FP	FN	Precision	Recall	*F*-score	Nominal *p*-value	Adjusted *p*-value
Delay	HRNN	5	3.9	9.3	6.0	0.32	0.40	0.35		
		10	7.5	20.1	11.8	0.28	0.39	0.32		
		20	11.6	43.9	28.5	0.21	0.29	0.24		
		30	19.1	64.9	39.2	0.22	0.33	0.26		
Delay	HCC-CLINDE	5	3.2	3.1	6.7	0.52	0.33	0.404	0.52	1
		10	7.7	6.2	11.6	0.57	0.40	0.47	0.06	0.24
		20	11.3	19.1	28.8	0.38	0.28	0.32	0.22	0.88
		30	17.0	20.5	41.3	0.46	0.29	0.35	0.23	0.92
Effect	HRNN	5	4.5	6.3	6.3	0.44	0.43	0.43		
		10	8.9	16.2	10.7	0.36	0.46	0.40		
		20	13.0	38.7	27.7	0.25	0.32	0.28		
		30	20.2	60.2	38.6	0.25	0.346	0.29		
Effect	HCC-CLINDE	5	4.0	2.3	5.9	0.67	0.42	0.51	0.32	1
		10	7.9	6.0	11.4	0.58	0.41	0.48	0.33	1
		20	11.5	18.9	28.6	0.39	0.29	0.33	0.44	1
		30	17.4	20.1	40.9	0.47	0.29	0.36	0.31	1

The *p*-values are for how the Delay and Effect *F*-scores of HCC-CLINDE method compare with HRNN. *P* is the number of genes for each of the networks. The variance of the noise is equal to 1

**Fig. 9:**
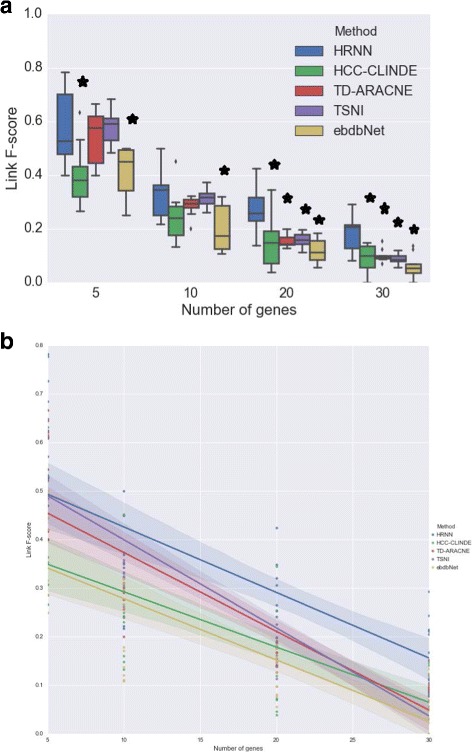
Effect of number of genes on Link *F*-score for case of non-linearity among gene interactions. For a chosen number of genes, 10 GRNs are randomly generated where *Var*(*noise*)=1. **a** Box plot of the Link *F*-score versus number of genes. ⋆ adjusted *p*-value ≤0.05**b** Linear regression model of the Link *F*-score versus number of genes

**Fig. 10:**
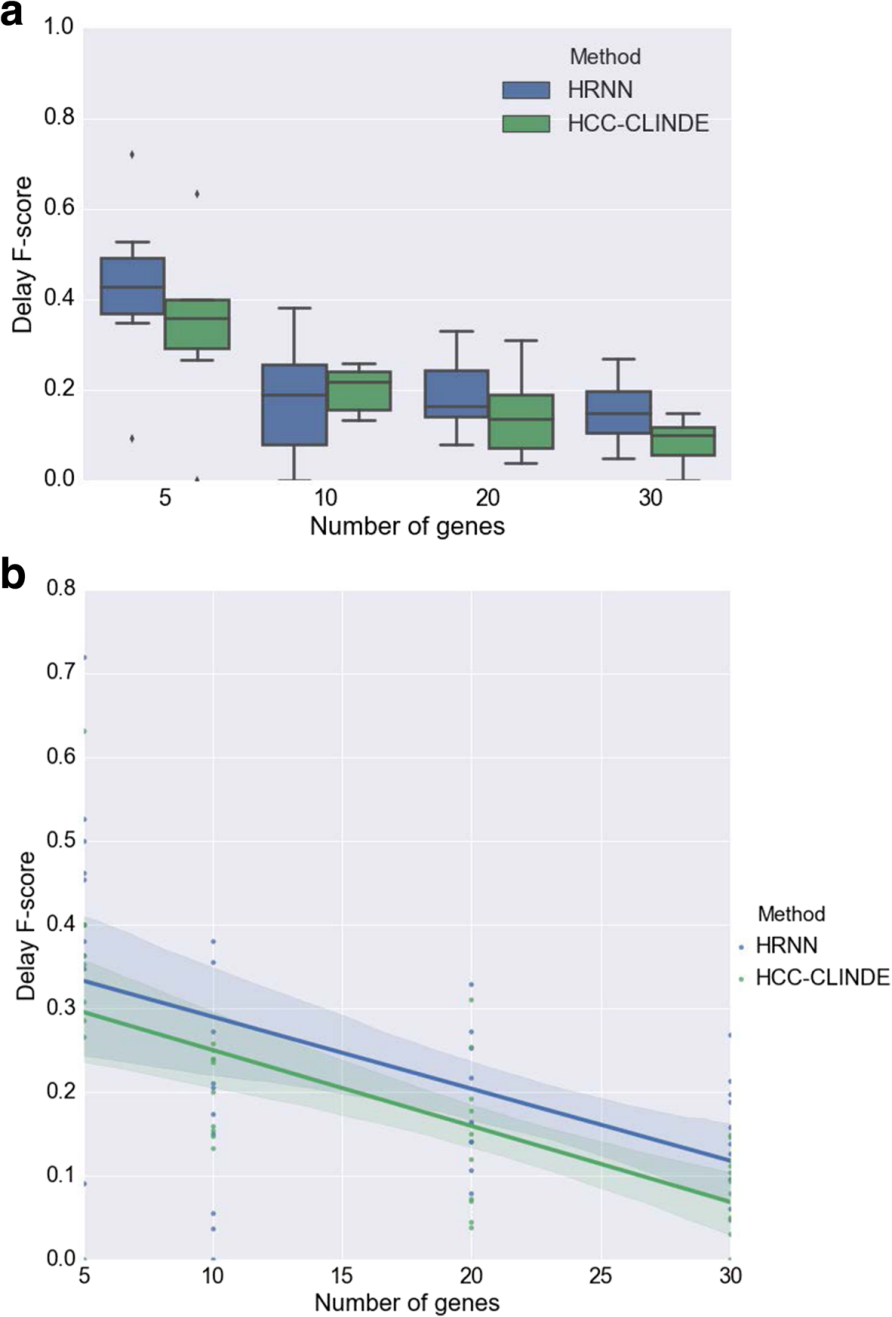
Effect of number of genes on Delay *F*-score for case of non-linearity among gene interactions. For a chosen number of genes, 10 GRNs are randomly generated where *Var*(*noise*)=1. **a** Box plot of the Delay *F*-score versus number of genes. ⋆ adjusted *p*-value ≤0.05**b** Linear regression model of the Delay *F*-score versus number of genes

**Fig. 11:**
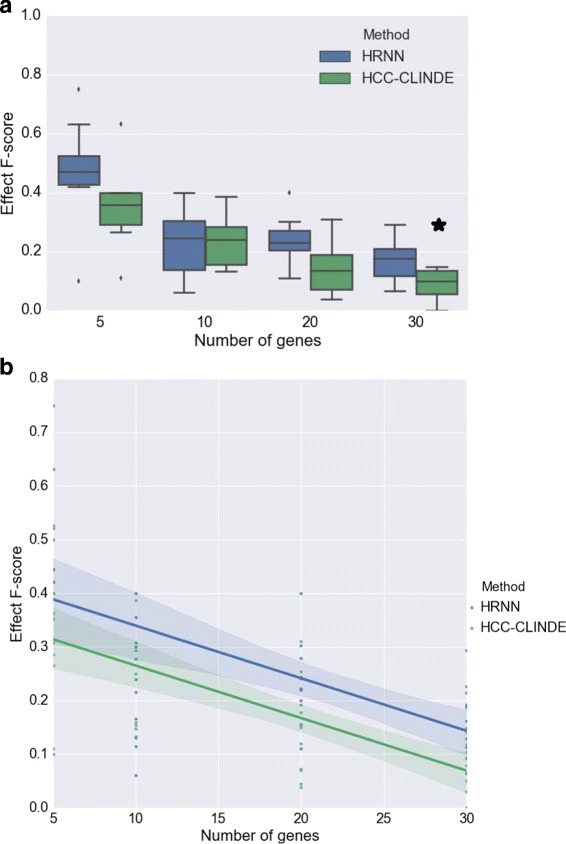
Effect of number of genes on Effect *F*-score for case of non-linearity among gene interactions. For a chosen number of genes, 10 GRNs are randomly generated where *Var*(*noise*)=1. **a** Box plot of the Effect *F*-score versus number of genes. ⋆ adjusted *p*-value ≤0.05**b** Linear regression model of the Effect *F*-score versus number of genes

In the next step for testing synthetic data, we considered a more realistic scenario where the gene expression values are generated from nonlinear models. For 10 different randomly generated networks, the effect of the number of genes in the accuracy of GRN reconstruction of HRNN was compared with other methods. Figures 9, 10 and 11 compare the accuracy of Link *F*-score, Delay *F*-score and Effect *F*-score for different number of genes in the network respectively. The variance of noise in these experiments is the same, and is equal to (*σ* ^2^=1). The linear regression models between *F*-score and number of genes, shown in part *b* of these figure. *R*-squared and *p*-values of the linear regression models in Figs. 9(b), 10(b) and 11(b) are stated in Table 4.

**Table 4:** The statistical properties of the linear regression models fitted on the *F*-score values with respect to the number of genes

	Method	*R*-squared	*p*-value
Link	HRNN	0.558	2.9×10^−8^
	HCC-CLINDE	0.56	2.3×10^−8^
	TD-ARACNE	0.74	7.0×10^−13^
	TSNI	0.81	1.2×10^−15^
	ebdbNet	0.64	3.8×10^−10^
Delay	HRNN	0.28	4.0×10^−4^
	HCC-CLINDE	0.43	3.4×10^−6^
Effect	HRNN	0.347	6.2×10^−5^
	HCC-CLINDE	0.49	3.4×10^−7^

Gene expression data is generated from a non-linear model

Tables 5 and 6 include the average of the TP, FP, FN, Precision, Recall and *F*-score in term of Link, Delay and Effect criterion for 10 independent runs of the methods and different number of genes in the network. For a selected number of genes in these tables, a *t*-test performed on *F*-score values. If the corresponding adjusted *p*-value is less than 0.05, the null hypothesis is rejected, meaning that mean of *F*-score of HRNN is significantly different from other method. The results show that the proposed HRNN works better than HCC-CLINDE, TD-ARACNE, TSNI and ebdbNet for cases of non-linearity among gene interactions in bigger networks. The accuracy of HCC-CLINDE for finding the correct link drops significantly in case of non-linearity between the genes in comparison to the the linear relationships.

**Table 5:** The effect of network size on GRN reconstruction in case of non-linearity between the genes. Results are the average of the accuracy in terms of the Link criteria for GRN reconstruction of 10 different randomly generated synthetic networks

Methods	*P*	TP	FP	FN	Precision	Recall	*F*-score	Nominal *p*-value	Adjusted *p*-value
HRNN	5	5.6	3.4	4.7	0.62	0.54	0.57		
	10	6.4	12.8	12.6	0.33	0.33	0.33		
	20	11.2	30.5	28.4	0.26	0.28	0.27		
	30	10.9	50.3	46.1	0.18	0.19	0.18		
TD-ARACNE	5	6.60	6.9	3.7	0.50	0.64	0.54	0.60	1
	10	9.3	35.7	9.7	0.21	0.50	0.28	0.16	0.64
	20	14.5	129.5	25.1	0.10	0.37	0.15	3.0×10^−4^	1.2×10^−3^
	30	12	162	45	0.07	0.20	0.09	2.0×10^−5^	8.0×10^−5^
HCC-CLINDE	5	3.1	1.8	7.2	0.65	0.29	0.39	7.0×10^−4^	2.8×10^−3^
	10	3.3	4.4	15.7	0.46	0.17	0.24	0.05	0.2
	20	4.1	9.4	35.5	0.29	0.10	0.15	0.007	0.028
	30	3.4	13.0	53.6	0.21	0.05	0.09	0.002	0.008
TSNI	5	10.3	14.7	0.0	0.41	1	0.58	0.91	1
	10	16.9	71.1	2.1	0.19	0.89	0.31	0.60	1
	20	17.6	160.4	22.0	0.09	0.44	0.16	4.0×10^−4^	1.6×10^−3^
	30	10	158	47	0.06	0.17	0.08	2.0×10^−4^	8.0×10^−4^
ebdbNet	5	4.4	6.1	5.9	0.43	0.43	0.41	0.008	0.032
	10	4.8	20.2	14.2	0.19	0.25	0.20	0.004	0.016
	20	6.2	55.80	33.4	0.10	0.15	0.12	5.0×10^−5^	2.0×10^−4^
	30	3.8	59.2	53.2	0.06	0.06	0.06	7.9×10^−5^	3.1×10^−4^

The *p*-values are for how the Link *F*-scores of other methods compare with HRNN. *P* is the number of genes for each of the networks. The variance of the noise is equal to 1

**Table 6:** The effect of network size on GRN reconstruction in case of non-linearity between the genes. Results are the average of the accuracy in terms of the Delay and Effect criterion for GRN reconstruction of 10 different randomly generated synthetic networks

	Methods	*P*	TP	FP	FN	Precision	Recall	*F*-score	Nominal *p*-value	Adjusted *p*-value
Delay	HRNN	5	4.7	7.0	5.6	0.40	0.45	0.42		
		10	3.9	19.0	15.1	0.17	0.19	0.18		
		20	8.0	37.8	31.6	0.17	0.20	0.18		
		30	9.0	55.4	48.0	0.14	0.16	0.15		
Delay	HCC-CLINDE	5	2.6	2.3	7.7	0.57	0.25	0.34	0.25	1
		10	2.6	2.3	7.7	0.57	0.25	0.34	0.69	1
		20	3.9	9.6	35.7	0.28	0.09	0.14	0.27	1
		30	3.3	13.1	53.7	0.21	0.05	0.08	0.03	0.12
Effect	HRNN	5	4.8	4.3	6.0	0.52	0.44	0.47		
		10	4.6	15.0	14.5	0.23	0.23	0.23		
		20	9.8	32.3	29.9	0.23	0.24	0.23		
		30	9.9	51.7	47.3	0.16	0.17	0.17		
Effect	HCC-CLINDE	5	2.7	2.2	7.6	0.59	0.26	0.35	0.08	0.32
		10	3.1	4.6	15.9	0.43	0.16	0.23	0.95	1
		20	3.9	9.6	35.7	0.28	0.09	0.14	0.02	0.08
		30	3.4	13.0	53.6	0.21	0.05	0.09	0.01	0.04

The *p*-values are for how the Delay and Effect *F*-scores of HCC-CLINDE method compare with HRNN. *P* is the number of genes for each of the networks. The variance of the noise is equal to 1

**Fig. 12:**
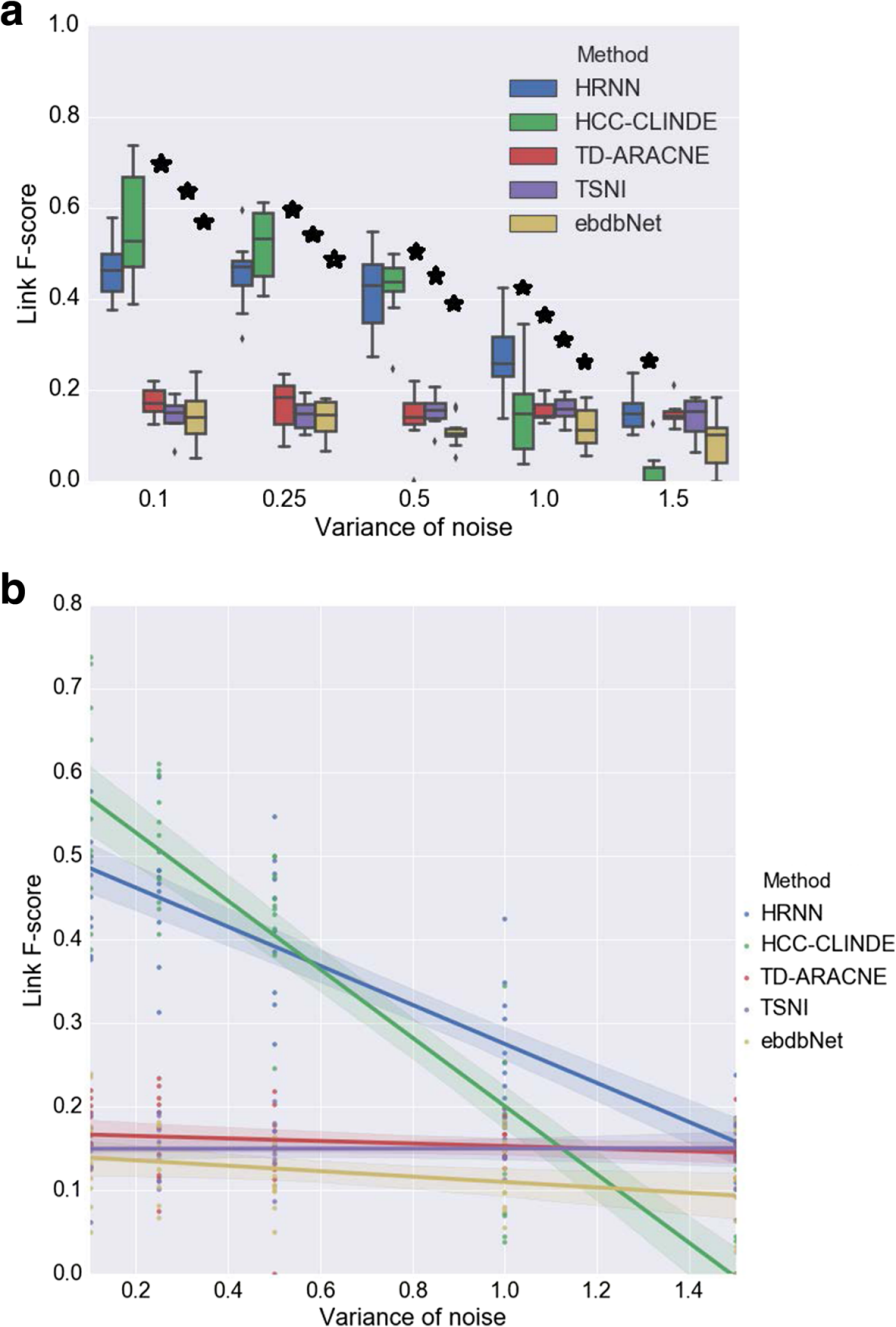
Effect of noise level on Link *F*-score for case of non-linearity among gene interactions. For a chosen variance of the noise, 10 GRNs are randomly generated where number of genes is 20. **a** Box plot of the Link *F*-score versus variance of the noise. ⋆ adjusted *p*-value ≤0.05**b** Linear regression model of the Link *F*-score versus variance of the noise

**Fig. 13:**
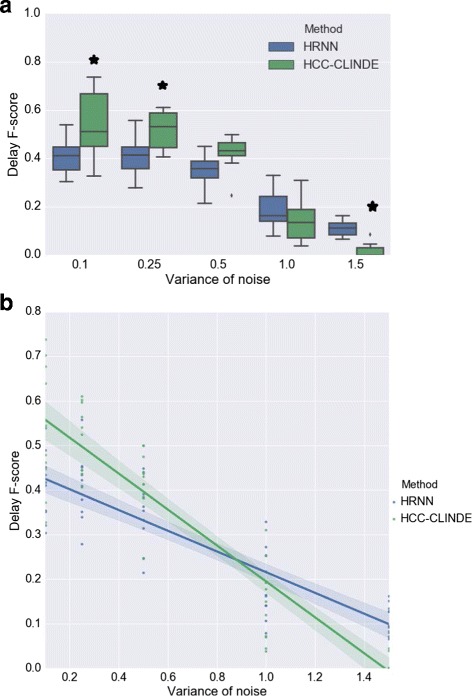
Effect of noise level on Delay *F*-score for case of non-linearity among gene interactions. For a chosen variance of the noise, 10 GRNs are randomly generated where number of genes is 20. **a** Box plot of the Delay *F*-score versus variance of the noise. ⋆ adjusted *p*-value ≤0.05**b** Linear regression model of the Delay *F*-score versus variance of the noise

**Fig. 14:**
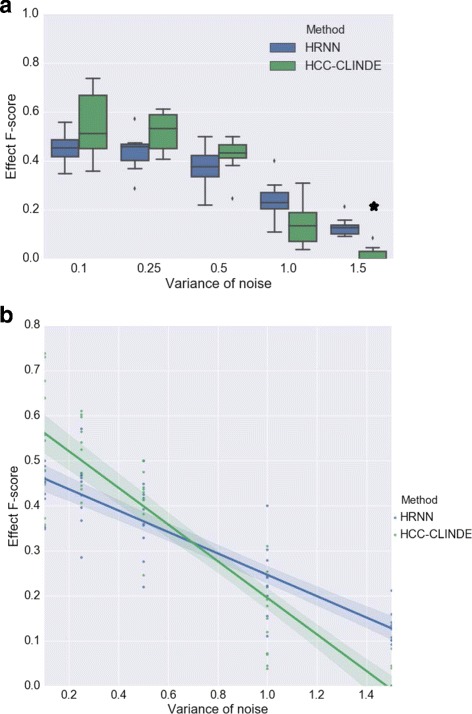
Effect of noise level on Effect *F*-score for case of non-linearity among gene interactions. For a chosen variance of the noise, 10 GRNs are randomly generated where number of genes is 20. **a** Box plot of the Effect *F*-score versus variance of the noise. ⋆ adjusted *p*-value ≤0.05**b** Linear regression model of the Effect *F*-score versus variance of the noise

In another simulation, the effect of noise in non-linear models is examined. In synthetic non-linear networks with 20 genes, the level of noise is changed (*σ* ^2^∈{0.1,0.25,0.5,1.0,1.5}). Figures 12, 13 and 14 show the results of GRN reconstruction of HRNN in comparison to TD-ARACNE, HCC-CLINDE, TSNI and ebdbNet in the forms of box plot and linear regression model and in terms of Link, Delay and Effect *F*-scores respectively. *R*-squared and *p*-values of the linear regression models in Figs. 12(b), 13(b) and 14(b) are stated in Table 7. Tables 8 and 9 include the average of the TP, FP, FN, Precision, Recall, *F*-score, nominal and adjusted *p*-value for 10 independent runs of the methods and different levels of the noise. HRNN is tested multiple times for five different noise levels of gene measurements, to obtain the adjusted *p*-values, the nominal *p*-values are multiplied by five. Figure 12 show that the accuracy of our proposed method in terms of Link *F*-score is often higher than HCC-CLINDE, TD-ARACNE, TSNI and ebdbNet. Figures 13 and 14 show that the HRNN and HCC-CLINDE are competitive in terms of Delay and Effect *F*-scores.

**Table 7:** The statistical properties of the linear regression models fitted on the *F*-score values with respect to the noise level

	Method	*R*-squared	*p*-value
Link	HRNN	0.74	5.5×10^−16^
	HCC-CLINDE	0.84	2.2×10^−21^
	TD-ARACNE	0.03	0.19
	TSNI	4.0×10^−5^	0.96
	ebdbNet	0.10	0.02
Delay	HRNN	0.74	5.1×10^−16^
	HCC-CLINDE	0.83	1.4×10^−20^
Effect	HRNN	0.76	6.9×10^−17^
	HCC-CLINDE	0.84	7.3×10^−21^

Gene expression data is generated from a non-linear model

**Table 8:** The effect of noise level on GRN reconstruction in case of non-linearity between the genes. Results are the average of accuracy of the Link criteria for GRN reconstruction of 10 different randomly generated synthetic networks

Methods	*σ* ^2^	TP	FP	FN	Precision	Recall	*F*-score	Nominal *p*-value	Adjusted *p*-value
HRNN	0.1	20.2	28.3	19.0	0.41	0.51	0.46		
	0.25	19.9	26.7	20.6	0.42	0.49	0.45		
	0.5	17.9	28.4	21.3	0.38	0.45	0.41		
	1	11.2	30.5	28.4	0.26	0.28	0.27		
	1.5	5.9	32.2	33.6	0.15	0.14	0.15		
TD-ARACNE	0.1	13.5	102.5	25.7	0.12	0.34	0.17	1.9×10^−10^	9.5×10^−10^
	0.25	14.7	107.3	25.8	0.11	0.36	0.16	1.4×10^−8^	7.0×10^−8^
	0.5	12.0	102.0	27.2	0.09	0.30	0.13	1.3×10^−7^	6.5×10^−7^
	1	14.5	129.5	25.1	0.10	0.37	0.15	3.0×10^−4^	1.5×10^−3^
	1.5	12.2	115.8	27.3	0.11	0.31	0.14	0.86	1
HCC-CLINDE	0.1	19.0	9.5	20.2	0.66	0.48	0.56	0.04	0.2
	0.25	18.0	10.3	22.5	0.63	0.44	0.52	0.08	0.4
	0.5	12.8	8.4	26.4	0.61	0.33	0.42	0.78	1
	1	4.1	9.4	35.5	0.29	0.10	0.15	0.01	0.05
	1.5	0.5	7.3	39.0	0.06	0.01	0.02	1.2×10^−6^	6.0×10^−6^
TSNI	0.1	14.1	139.9	25.1	0.09	0.36	0.14	6.2×10^−11^	3.1×10^−10^
	0.25	13.6	128.4	26.9	0.09	0.33	0.14	7.2×10^−10^	3.6×10^−9^
	0.5	15.3	138.7	23.9	0.09	0.38	0.15	4.2×10^−8^	2.1×10^−7^
	1	17.6	160.4	22.0	0.09	0.44	0.16	4.8×10^−4^	2.4×10^−3^
	1.5	15.1	150.9	24.4	0.08	0.37	0.14	0.63	1
ebdbNet	0.1	7.8	56.2	31.4	0.12	0.20	0.14	1.5×10^−9^	7.5×10^−9^
	0.25	7.7	58.3	32.8	0.11	0.18	0.13	8.9×10^−10^	4.4×10^−9^
	0.5	5.2	48.8	34.0	0.09	0.13	0.10	4.4×10^−9^	2.2×10^−8^
	1	6.2	55.8	33.4	0.10	0.15	0.12	5.0×10^−5^	2.5×10^−4^
	1.5	5.2	54.8	34.3	0.07	0.13	0.09	0.02	0.1

The *p*-values are for how the Link *F*-scores of other methods compare with HRNN. *σ* ^2^ is variance of the noise. Networks include 20 genes

**Table 9:** The effect of noise level on GRN reconstruction in case of non-linearity between the genes

	Methods	*σ* ^2^	TP	FP	FN	Precision	Recall	*F*-score	Nominal *p*-value	Adjusted *p*-value
Delay	HRNN	0.1	18.7	34.2	20.5	0.35	0.47	0.41		
		0.25	18.5	31.9	22.0	0.36	0.45	0.41		
		0.5	15.6	34.4	23.6	0.31	0.39	0.34		
		1	8.0	37.8	31.6	0.17	0.20	0.18		
		1.5	4.5	37.3	35.0	0.11	0.11	0.11		
Delay	HCC-CLINDE	0.1	18.3	10.2	20.9	0.64	0.46	0.53	0.01	0.05
		0.25	17.9	10.4	22.6	0.63	0.44	0.51	0.005	0.025
		0.5	12.7	8.5	26.5	0.60	0.32	0.42	0.03	0.15
		1	3.9	9.6	35.7	0.28	0.09	0.14	0.27	1
		1.5	0.4	7.4	39.1	0.05	0.01	0.01	3.9×10^−6^	1.9×10^−5^
Effect	HRNN	0.1	19.8	29.3	19.8	0.40	0.50	0.44		
		0.25	19.2	27.6	21.9	0.41	0.46	0.43		
		0.5	16.1	30.7	23.5	0.34	0.40	0.37		
		1	9.8	32.3	29.9	0.23	0.24	0.23		
		1.5	5.1	33.3	34.5	0.13	0.12	0.13		
Effect	HCC-CLINDE	0.1	18.5	10.0	20.7	0.64	0.47	0.54	0.06	0.3
		0.25	18.0	10.3	22.5	0.63	0.44	0.52	0.02	0.1
		0.5	12.7	8.5	26.5	0.60	0.32	0.42	0.16	0.8
		1	3.9	9.6	35.7	0.28	0.09	0.14	0.02	0.1
		1.5	0.4	7.4	39.1	0.05	0.01	0.01	3.4×10^−7^	1.7×10^−6^

Results are the average of accuracy of the Delay and Effect criterion for GRN reconstruction of 10 different randomly generated synthetic networks. The *p*-values are for how the Delay and Effect *F*-scores of HCC-CLINDE method compare with HRNN. *σ* ^2^ is variance of the noise. Networks include 20 genes

### Real-life biological data of Saccharomyces cerevisiae (IRMA)

In order to validate the performance of the proposed method on real-life biological GRNs, we considered a recent significant contribution to systems biology reported in [36] where the authors built a synthetic network, called IRMA, of the yeast organism *Saccharomyces cerevisiae*. The researchers tested transcription of network genes by culturing cells in presence of galactose and glucose. This is one of the first attempts at building a reference data set, having a fairly true table of regulations [8, 23]. The regulatory network includes five genes. It is negligibly affected by endogenous genes. Two sets of gene profiles called Switch ON and Switch OFF were provided, each containing 16 and 21 time points. The former corresponds to the shifting of the growing cells from glucose to the galactose medium; the latter corresponds to the reverse shift. Due to the lack of stimulus, reconstruction of the GRN from the Switch OFF dataset is difficult [8, 23].

**Fig. 15:**
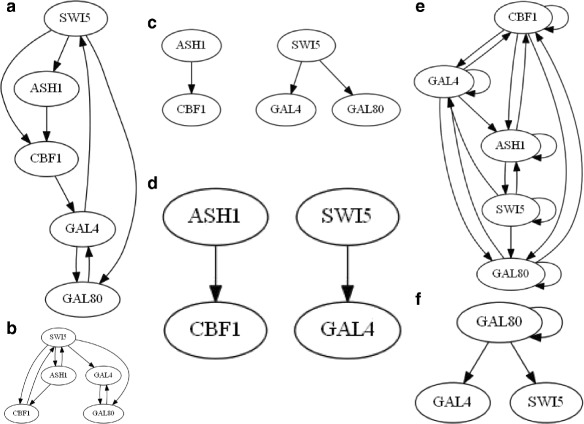
GRN reconstruction of real biological system of IRMA. **a** True regulations **b** HRNN **c** TD-ARACNE **d** HCC-CLINDE **e** TSNI **f** ebdbNet

The performance comparisons among various methods for the IRMA ON dataset are shown in Fig. 15 and Table 10. In Fig. 15(a), the true IRMA network is shown. Figure 15(b) displays the GRN obtained by the proposed method. Figure 15(c-f) present the GRN reconstructions by TD-ARACNE, HCC-CLINDE, TSNI and ebdbNet obtained with default parameters. In Table 10, TP, FP, FN, precision, recall and *F*-score values are also compared. The proposed HRNN finds the true regulations of ASH1 by SWI5, CBF1 by ASH1, GAL80 by SWI5, GAL4 by GAL80, GAL80 by GAL4 and CBF1 by SWI5. The regulation of SWI5 by GAL4 and regulation of GAL4 by CBF1 are not found. The method finds three regulations (regulations of SWI5 by CBF1, SWI5 by ASH1 and SWI5 by GAL4) that are not in the true network of IRMA. Among the eight connections in the true network, TD-ARACNE finds two correct regulations. The HCC-CLINDE method found one true regulation and one false regulation. Also, HCC-CLINDE finds the regulations of ASH1 and SWI5 by a hidden common cause which is not reported in the actual GRN of the IRMA. Results show higher accuracy in the proposed HRNN approach for finding the regulatory interactions between the genes in comparison to the other two approaches.

**Table 10:** Comparison of results for GRN reconstructions of IRMA

Methods	TP	FP	FN	Precision	Recall	*F*-score
HRNN	6	3	2	0.66	0.75	0.70
TD-ARACNE	2	1	6	0.66	0.25	0.36
HCC-CLINDE	1	3	7	0.25	0.12	0.16
TSNI	6	12	2	0.33	0.75	0.46
ebdbNet	1	2	7	0.33	0.12	0.18

## Conclusions

In this study, we developed and implemented a hierarchical recurrent neural network (HRNN) approach to identify time-delayed regulatory interactions of genes. The designed HRNN facilitates capturing the paths with different lengths from the leaf nodes in the network to the target node. Hierarchy and non-linearity in the network and the allowance for recurrent connections in HRNN provide an effective capability for modeling the temporal patterns of gene expression. Furthermore, partial connectivity of the nodes aids in finding the limited set of genes which regulate the target gene over different time delays. The proposed method outperformed TD-ARACNE, HCC-CLINDE, TSNI and ebdbNet in terms of reconstructing small and medium size networks having non-linearity and high levels of noise for measurement data.

## Abbreviations

GA: Genetic algorithm
GRN: Gene regulatory network
HRNN: Hierarchical recurrent neural network
